# MiR-135b-5p and MiR-499a-3p Promote Cell Proliferation and Migration in Atherosclerosis by Directly Targeting MEF2C

**DOI:** 10.1038/srep12276

**Published:** 2015-07-17

**Authors:** Zhiliang Xu, Yeming Han, Jiying Liu, Fan Jiang, Huili Hu, Yan Wang, Qiji Liu, Yaoqin Gong, Xi Li

**Affiliations:** 1 Key Laboratory of Experimental Teratology, Ministry of Education Institute of Medical Genetics, School of Medicine, Shandong University Jinan, Shandong 250012, China; 2 Department of Cardiology, Shandong University Qilu Hospital, Jinan, Shandong 250012, China; 3 Department of Interventional Treatment, Shandong Provincial Hospital Affiliated to Shandong University, Jinan, Shandong 250021, China; 4 Key Laboratory of Cardiovascular Remodeling and Function Research, Ministry of Education and Ministry of Health, Shandong University Qilu Hospital, Jinan, Shandong 250012, China

## Abstract

Proliferation and migration of endothelial cells (ECs) and vascular smooth muscle cells (VSMCs) are critical processes involved in atherosclerosis. Recent studies have revealed that microRNAs (miRNAs) can be detected in circulating blood with a stable form and the expression profiles differ in many cellular processes associated with coronary artery disease (CAD). However, little is known about their role, especially serum-derived miRNAs, in ECs and VSMCs phenotype modulation during atherosclerosis. We compared the miRNA expressions in serum samples from 13 atherosclerotic CAD patients and 5 healthy control subjects and identified 36 differentially expressed miRNAs. The expression of selected miRNAs (miR-135b-5p and miR-499a-3p) was further validated in 137 serum samples. Interestingly, miR-135b-5p and miR-499a-3p directly regulated a common target gene: myocyte enhancer factor 2C (*MEF2C*) which plays an important role in modulating cell phenotype of cardiovascular systems. Furthermore, our results indicated that the 2 elevated miRNAs could jointly promote ECs and VSMCs proliferation and migration by repressing *MEF2C* expression. Together, our findings demonstrated a serum-based miRNA expression profile for atherosclerotic CAD patients, potentially revealing a previously undocumented mechanism for cell proliferation and migration mediated by miR-135b-5p and miR-499a-3p, and might provide novel insights into the role of circulating miRNAs in atherosclerosis pathogenesis.

Atherosclerosis, a chronic disease characterized by the accumulation of lipids and fibrous elements in the large arteries, constitutes the single most important contributor to coronary artery disease (CAD), a leading cause of morbidity and mortality worldwide^1^. In the pathogenesis of atherosclerosis, dysfunction of endothelial cells has been implicated as an early step followed by invasion of proinflammatory cells, and proliferation and migration of smooth muscle cells^1^.

MicroRNAs (miRNAs) are noncoding RNA molecules that regulate the post-transcription expression of target mRNAs^2^. Recent studies have demonstrated that miRNAs are remarkably stable in circulating blood^3^ and the expression profiles are quite representative in CAD^4^,^5^,^6^. For example, plasma-isolated miR-126, -17, -92a, and -155 are shown to be significantly downregulated in CAD patients^4^. Peripheral blood miR-21, -122, and -130a were significantly increased whereas miR-92a, -126, and -222 were decreased in atherosclerotic CAD patients^5^. Hoekstra *et al.* have reported that miR-198 and -370 are upregulated and miR-147 is downregulated in the peripheral blood mononuclear cells of individuals with CAD^6^. In addition, circulating miR-146a /146b are shown to be significantly upregulated in CAD patients^7^. The importance of circulating miRNAs in atherosclerotic CAD is starting to be recognized^8^,^9^, but the effects of atherosclerosis exerted on the expression of serum-isolated miRNAs are unknown. More importantly, the potential biological functions of serum-isolated miRNAs in critical cellular processes associated with atherosclerosis remain largely unknown.

This study is directed toward the issue and a miRNA array was used to comparatively analyze serum-based miRNA expression profile of CAD patients with atherosclerosis and corresponding healthy controls. Furthermore, several differentially expressed miRNAs were selected and tested. Among all the validated miRNAs, miR-135b-5p and miR-499a-3p are of particular interest because both miRNAs were shown to regulate the same target gene: myocyte enhancer factor 2C (*MEF2C*). MEF2C, as a member of the myocyte-enhancer factor 2 family of transcription factors^10^,^11^, shows homology in a MADS (MCM1, Agamous, Deficiens, serum response factor) box and an adjacent motif known as the MEF2 domain^12^. Notably, MEF2C is mainly involved in cell proliferation, migration and cellular homeostasis in cardiovascular systems which are known to play important roles in modulating cell phenotype^13^,^14^,^15^.

Here, the experimental evidences showed that the two elevated miRNAs (miR-135b-5p and miR-499a-3p) repressed target *MEF2C* gene expression. Furthermore, in light of the important role of MEF2C in cell phenotypic modulation, the enhanced proliferation and migration in response to the miRNAs-mediated *MEF2C* suppression were demonstrated in endothelial cells (ECs) and vascular smooth muscle cells (VSMCs). Moreover, immunohistochemical staining showed that MEF2C protein was downregulated in human atherosclerotic plaque compared with the corresponding vascular wall. Given that miR-135b-5p and miR-499a-3p are mainly found in microvesicles (MVs)^16^, our findings are discussed with regard to the known pathophysiological function of MV-associated miRNAs accumulated in atherosclerotic plaques^17^. Collectively, the present studies identified two elevated serum-isolated miRNAs (miR-135b-5p and miR-499a-3p) of atherosclerotic CAD patients and might offer novel insight into the pathological relationships between circulating miRNAs and atherosclerosis.

## Results

### Patient Characteristics

One hundred and fifty five subjects were studied. The microarray cohort of subjects included 13 atherosclerotic patients and 5 healthy control subjects. The clinical characteristics of this population are summarized in Supplementary Table S1. Furthermore, we studied a second group composed of 77 atherosclerotic CAD patients and 45 healthy control subjects for independent validation. Their characteristics are summarized in Supplementary Table S2. In addition, 15 non-atherogenic cardiovascular patients (3 with cardiogenic shock, 5 with heart failure, and 7 with ventricular arrhythmia; 10 females and 5 males; mean age 43.36 ± 8.12 years) served as another control group in the independent validation of miR-135b-5p and miR-499a-3p expressions.

### MiRNA Profiles in Atherosclerotic CAD Patients Versus That of Healthy Volunteers

**Figure 1:**
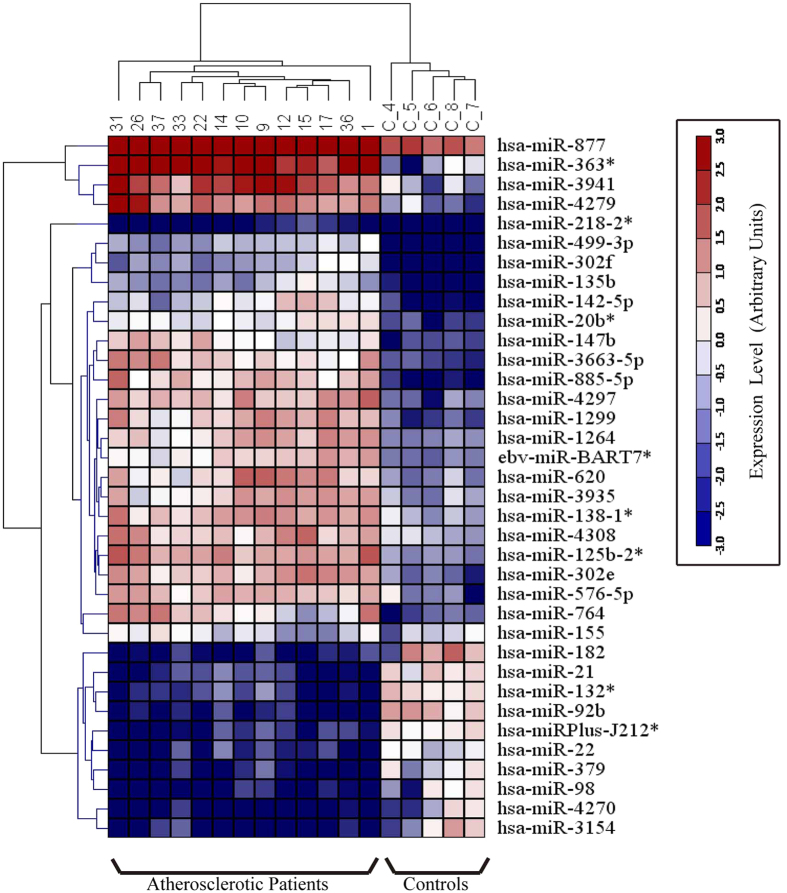
Heat map of microRNA (miRNA) microarray expression data from serum samples of atherosclerotic patients (n = 13) and healthy control subjects (n = 5). The expression of miRNA is hierarchically clustered on the *y* axis, and atherosclerotic serum samples or healthy control serum samples are hierarchically clustered on the *x* axis. The relative miRNA expression is depicted according to the color scale shown on the right. Red indicates upregulation; blue, downregulation. Numbers (1, 9, 10, 12, 14, 15, 17, 22, 26, 31, 33, 36, 37) indicate atherosclerotic patients’ samples; numbers (C-4, 5, 6, 7, 8) indicate healthy control samples.

To identify aberrantly circulating miRNAs during atherosclerosis, a serum-based miRNA expression was performed in 13 atherosclerotic CAD patients and 5 healthy donors using the miRCURY LNA Array (version 11.0) system (GEO Submissions Number: GSE 53675). The levels of circulating miRNAs differed significantly between patients and healthy controls (Fig. 1). Of 1,700 miRNAs detected on the microarray, 36 were found to be differentially expressed in the patients compared with that of the controls (P < 0.05); 26 miRNAs were increased and 10 were decreased in the patient samples compared with those in the control samples. Quantification revealed that multiple miRNAs were significantly elevated in the patients compared with that in the healthy controls. According to their known and postulated functions, 13 miRNAs were selected and further tested. The miRNA names, fold changes, and *P* values in the microarray cohort are presented in Table 1. In addition, the relative expressions of the 13 miRNAs in atherosclerotic patients compared with that in the healthy controls were shown in Supplementary Fig. S1 and the relative expressions of the other 26 miRNAs were presented in Supplementary Fig. S2.

### Validation of MiRNAs Expressions

**Figure 2:**
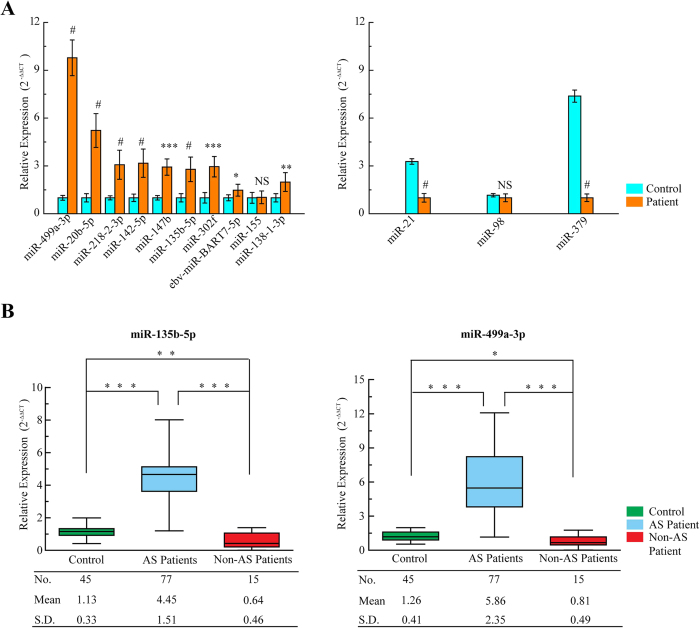
Validation of miRNAs expressions by qRT-PCR. **(A)** The microarray cohort included 13 atherosclerotic patients and 5 healthy control subjects. The relative expression of 13 miRNAs was normalized to expression of the internal control (miR-93). The *P* values were calculated by 2-sided Student *t* test. *P < 0.05; **P < 0.01; ***P < 0.001; ^#^*P* < 0.0001. **(B)** qRT-PCR for miR-135b-5p and miR-499a-3p in an independent validation set of 77 atherosclerotic patients, 15 non-atherogenic cardiovascular patients and 45 healthy control subjects. The *P* values were calculated by 2-sided Student *t* test. *P < 0.05; **P < 0.01; ***P < 0.001.

To confirm the findings of the miRNA profile, the expression of these 13 dysregulated miRNAs was measured using qRT-PCR. The relative expression of each miRNA in atherosclerotic patients compared with that in the healthy controls was shown in Fig. 2A. The results demonstrated a 9.7-fold increase in miR-499a-3p (P < 0.0001) and a 2.7-fold increase in miR-135b-5p (P < 0.0001) in atherosclerotic CAD patients.

MiR-135b-5p and miR-499a-3p are of particular interest among all the validated miRNAs. MiR-499a-3p was the focus of this study because it had the highest expression level in atherosclerotic patients compared with that in healthy controls, both in the miRNA profile and quantitative PCR (Table 1 and Fig. 2A). Although miR-135b-5p is known to be associated with cancer^18^,^19^, recent work suggests a critical role for this miRNA regulated by the interleukin (IL)-1 receptor in mediating inflammatory responses^20^. Most importantly, computational analysis from several widely used mammalian target prediction programs, including miRBase, TargetScan, miRanda, and PicTar^21^,^22^,^23^, indicated that both miR-135b-5p and miR-499a-3p regulate several common target genes, which potentially contributes to impaired cell function in atherosclerosis.

To further validate the elevation of miR-135b-5p and miR-499a-3p in the atherosclerotic process, a series of independently validation cohorts were measured. One hundred and twenty-two serum samples were collected from 77 atherosclerotic CAD patients and 45 healthy control subjects. The clinical features of these subjects (Supplementary Table S2) did not significantly differ from that of the previous set in the miRNA profiles (Supplementary Table S1). As shown in Fig. 2B, the 2 miRNAs were found to be significantly upregulated in atherosclerotic patients compared with those in the healthy controls. Our results showed a 3.9-fold increase in miR-135b-5p and a 4.7-fold increase in miR-499a-3p expression in atherosclerotic CAD patients compared with those in healthy control subjects (both P < 0.001). To confirm the cardiac atherogenic ontogeny of the 2 miRNAs, miR-135b-5p and miR-499a-3p were also assessed in 15 non-atherogenic cardiovascular patients (3 with cardiogenic shock, 5 with heart failure, and 7 with ventricular arrhythmia). As shown in Fig. 2B, the results showed decreasing trends of miR-135b-5p and miR-499a-3p levels in non-atherogenic cardiovascular patients compared to those in healthy control subjects. In summary, these data demonstrated that circulating levels of miR-135b-5p and miR-499a-3p were significantly increased in CAD patients with atherosclerosis.

Additionally, we also analyzed the association of the two miRNAs with specific factors for atherosclerosis. Both miR-135b-5p and miR-499a-3p were significantly associated with age, stoke, beta-blockers, and aspirin (Supplementary Table S3). MiR-135b-5p was highly influenced by ACE inhibitors and statin treatment was significantly associated with increased levels of circulating miR-499a-3p (Supplementary Table S3); however, when only atherosclerotic patients were included in the analysis, hypertension was inversely correlated with the levels of circulating miR-499a-3p and none of the other factors remained a significant factor (Supplementary Table S4).

### *MEF2C* Is a Direct Target of Both MiR-135b-5p and MiR-499a-3p

**Figure 3:**
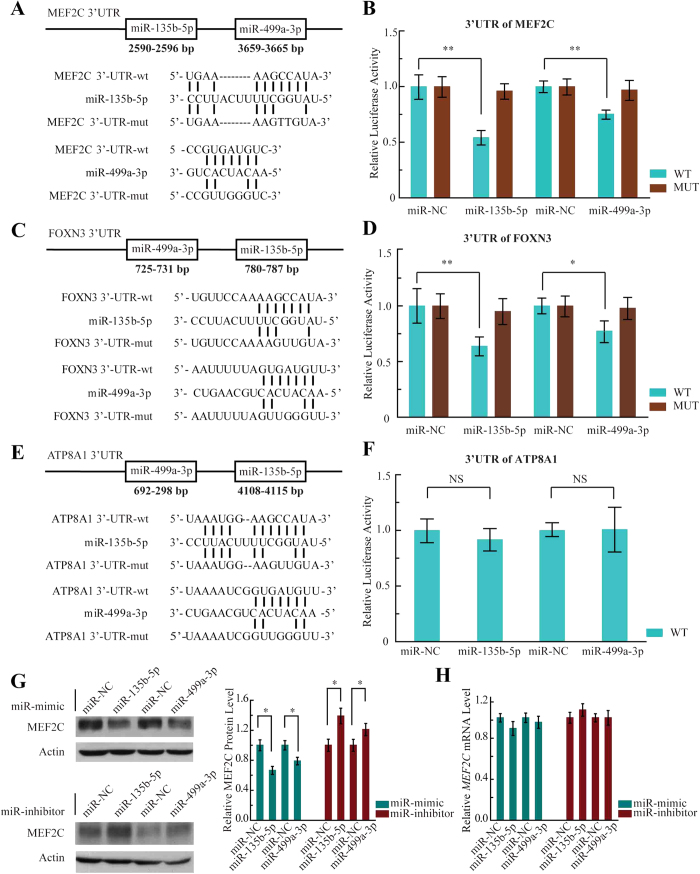
MiR-135b-5p and miR-499a-3p repressed *MEF2C* expression. **(A)** Predicted human *MEF2C*-encoded mRNA contains a 3′-UTR element that is partially complementary to the indicated miRNAs. Above: predicted target binding sites for miR-135b-5p and miR-499a-3p, respectively. Below: predicted duplex combination between the human *MEF2C* 3′-UTR-wt/mut and miR-135b-5p, *MEF2C* 3′-UTR-wt/mut and miR-499a-3p. **(B)** Luciferase activity of pmirGLO3-MEF2C-wt (WT) or pmirGLO-MEF2C-mut (MUT) in HEK293 cells after miR-135b-5p (left) and miR-499a-3p (right) transfections. Data are shown as means ± SD based on three independent experiments, **P < 0.01. **(C)** Predicted human *FOXN3*-encoded mRNA contains a 3′-UTR element that is partially complementary to the indicated miRNAs. Above: predicted target binding sites for miR-135b-5p and miR-499a-3p, respectively. Below: predicted duplex combination between the human *FOXN3* 3′-UTR-wt/mut and miR-135b-5p, *FOXN3* 3′-UTR-wt/mut and miR-499a-3p. **(D)** Luciferase activity of pmirGLO3-FOXN3-wt (WT) or pmirGLO-FOXN3-mut (MUT) in HEK293 cells after miR-135b-5p (left) and miR-499a-3p (right) transfections. Data are shown as means ± SD based on three independent experiments, *P < 0.05; **P < 0.01. **(E)** Predicted human *ATP8A1*-encoded mRNA contains a 3′-UTR element that is partially complementary to the indicated miRNAs. Above: predicted target binding sites for miR-135b-5p and miR-499a-3p, respectively. Below: predicted duplex combination between the human *ATP8A1* 3′-UTR-wt/mut and miR-135b-5p, *ATP8A1* 3′-UTR-wt/mut and miR-499a-3p. **(F)** Luciferase activity of pmirGLO3-ATP8A1-wt (WT) in HEK293 cells after miR-*1*35b-5p (left) and miR-499a-3p (right) transfections. Data are shown as means ± SD based on three independent experiments. **(G)** Western blot analysis of MEF2C expression in HEK293 cells transfected with the miRNA mimics and inhibitors (left) and compiled data of MEF2C protein level from three independent experiments (right). Columns, mean; Bars, ± SD; *P < 0.05. The full-length gels are presented in Supplementary Fig. S6. **(H)** Compiled data of MEF2C mRNA level analysis from three independent experiments after transfection of miRNAs is shown. Columns, mean; Bars, ± SD.

We next sought to identify direct target of miR-135b-5p and miR-499a-3p involved in atherosclerosis. We used the most commonly used bioinformatics prediction tools (TargetScan, PicTar, miRanda and miR database^21^,^22^,^23^ to identify the target. We limited our search to those targets that are commonly predicted at least by two programs, and also to those that showed species conservation among vertebrates. The potential targets of both miR-135b-5p and miR-499a-3p included *MEF2C*, *FOXN3* (Forkhead Box N3) and *ATP8A1* (ATPase, aminophospholipid transporter [APLT], class I, type 8A). On *MEF2C* 3′-UTR (4,363-bp length), target positions 2590–2596 was predicted for miR-135b-5p, and target positions 3659–3665 was predicted for miR-499a-3p (Fig. 3A). Next, a fragment of the 3′-UTR of *MEF2C* mRNA containing the wild type or mutated putative miR-135b-5p or miR-499a-3p corresponding binding sequence (Fig. 3A) was cloned into a luciferase reporter in a dual reporter construct. The luciferase reporter assay in HEK293 cells showed that the luciferase activity was decreased by 50% when cotransfected with miR-135b-5p and its corresponding pmirGLO-*MEF2C*-wt (WT), and 30% when cotransfected with miR-499a-3p and its corresponding pmirGLO-*MEF2C*-wt (WT), whereas no significant reduction in luciferase activity was observed when the cells were cotransfected with miR-NC (Fig. 3B*, WT*). The construct pmirGLO-*MEF2C*-mut (MUT) was then used to repeat the luciferase assay experiments in HEK293 cells, which showed that mutating the seed region for miR-135b-5p or miR-499a-3p in the pmirGLO-*MEF2C*-wt plasmid completely abrogated its regulatory activity (Fig. 3B*, MUT*). The results demonstrated that miR-135b-5p or miR-499a-3p might suppress *MEF2C* expression at corresponding binding sites at its 3′-UTR.

**Figure 4:**
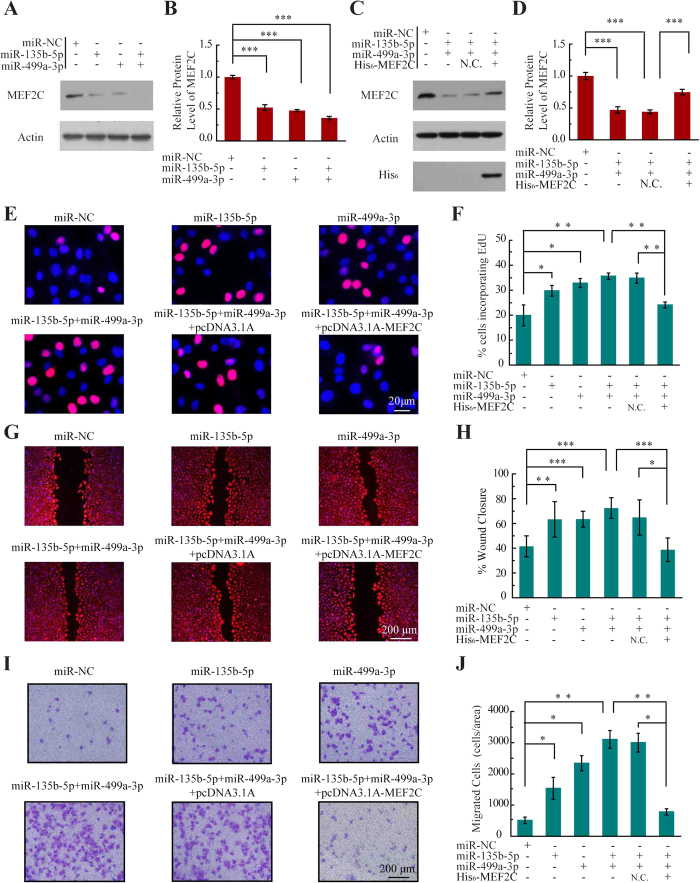
MiR-135b-5p and miR-499a-3p promote endothelial cell proliferation and migration. **(A)** Western blot analysis of MEF2C expression in HUVEC cells transfected with the indicated miRNAs mimics. The full-length gels are presented in Supplementary Fig. S7. **(B)** Compiled data from three independent experiments after transfection of miRNAs is shown. Columns, mean; Bars, ± SD; ***P < 0.001. **(C)** Western blot analysis of MEF2C expression in HUVEC cells transfected with the indicated miRNAs and pcDNA3.1A-MEF2C.The full-length gels are presented in Supplementary Fig. S7. **(D)** Compiled data from three independent experiments after transfection of miRNAs is shown. Columns, mean; Bars, ± SD; ***P < 0.001. **(E)** Photoimages of EdU incorporation assay of HUVECs that were treated with miRNAs or rescue plasmid. Results are representative data from three independent experiments. **(F)** Proliferation rates of HUVECs that were treated with miRNAs or rescue plasmid. Results are presented as means ± SD of three independent experiments. *P < 0.05; **P < 0.01. **(G)** Photoimages of scratch assay of HUVECs that were treated with miRNAs or rescue plasmid. Results are representative data from three independent experiments. **(H)** Migration rates of HUVECs that were treated with miRNAs or rescue plasmid. Results are presented as means ± SD of three independent experiments. *P < 0.05; **P < 0.01; ***P < 0.001. **(I)** Photoimages of transwell assay of HUVECs that were treated with miRNAs or rescue plasmid. Results are representative data from three independent experiments. **(J)** Migration rates of HUVECs that were treated with miRNAs or rescue plasmid. Results are presented as means ± SD of three independent experiments. *P < 0.05; **P < 0.01.

Furthermore, on each *FOXN3* and *ATP8A1* 3′-UTR, there are also target positions predicted for the two miRNAs (Fig. 3C,E). Then, we cloned the 3′-UTR fragments of *FOXN3* and *ATP8A1* containing the putative miR-135b-5p or miR-499a-3p corresponding binding sequence into a luciferase reporter construct, respectively. Indeed, overexpression of miR-135b-5p or miR-499a-3p significantly inhibited the luciferase activity of WT 3′-UTR of *FOXN3* (Fig. 3D), whereas there was no significant reduction in luciferase activity when cotransfected with miR-135b-5p (miR-499a-3p) and its corresponding pmirGLO-*ATP8A1*-wt (Fig. 4F). It will be interesting to study the regulations by which miR-135b-5p and miR-499a-3p target *FOXN3* gene in further studies; however, in the present study, we focused only on the investigation of the regulation of *MEF2C* for its important role in cell phenotypic modulation associated with cardiovascular systems. Moreover, an immunoblotting assay was conducted to verify the luciferase activity result. When mimics of miR-135b-5p or miR-499a-3p were transfected into HEK293 cells, MEF2C protein levels (but not mRNA) decreased compared to that in the negative control (Fig. 3G,H). Correspondingly, miR-135b-5p inhibitor or miR-499a-3p inhibitor could significantly increase MEF2C protein levels (Fig. 3G,H). Taken together, these results suggested that MEF2C is the valid target of miR-135-5p and miR-499a-3p.

### MiR-135b-5p and MiR-499a-3p Promoted Proliferation and Migration in HUVECs By Targeting MEF2C

Accumulating studies have demonstrated that circulating miRNAs can be actively taken up by cells, thus serving as signaling molecules mediating intercellular communication^24^,^25^,^26^. A survey of cell lines revealed that endogenous miR-135b-5p is highly enriched in muscle cells and T cells^27^,^28^ and endogenous miR-499a-3p is enriched in cardiac and skeletal muscle cells^4^, but *MEF2C* is mainly expressed in endothelial cells *in vivo*^15^ and *in vitro*^29^. In light of MEF2C roles in cell phenotypic modulation, we first evaluated the functional role of miR-135-5p and miR-499a-3p by measuring cell proliferation and migration in human umbilical vein endothelial cells (HUVECs).

HUVECs were first treated with mimics specific for miR-135b-5p, miR-499a-3p, miR-135b-5p + miR-499a-3p, or with miR-NC. Sixty-four percent of MEF2C protein expression was simultaneously decreased following transfection with (miR-135b-5p + miR-499a-3p) compared with 46% in cells transfected with miR-135b-5p or 53% in cells transfected with miR-499a-3p (Fig. 4A,B). To confirm that the 2 miRNAs regulate MEF2C levels, we tested whether MEF2C abundance could be rescued by coexpression with His_6_-tagged MEF2C. As shown in Fig. 4C,D, expression of His_6_-tagged MEF2C could rescue 77% of endogenous MEF2C.

To show the miRNAs-mediated MEF2C regulation was responsible for cell proliferation, incorporation of 5-ethynyl-2′-deoxyuridine (EdU) incorporation was monitored by immunofluorescence microscopy following the transfection of the HUVECs with miR-135b-5p, miR-499a-3p or pcDNA3.1-MEF2C. As shown in Fig. 4E,F, miR-135b-5p or miR-499a-3p accelerated proliferation compared with control mock-transfected cells. In particular, the proliferation was enhanced in both (miR-135b and miR-499a-3p) transfection cells (36.9% EdU-positive) compared to the single miRNA-transfected cells (27.9% and 30.7% EdU-positive, respectively), which is consistent with the immunoblotting results as shown in Fig. 4A. Next, the increase in cell proliferation caused by (miR-135b-5p + miR-499a-3p) transfection (36.9% EdU-positive) was offset in (miR-135b-5p + miR-499a-3p + pcDNA3.1-MEF2C) (21.1% EdU-positive) cells, demonstrating that the enhanced proliferation in the miRNAs transfection cells was indeed mediated by the downregulation of *MEF2C* (Fig. 4E,F).

To further determine whether the two miRNAs have an additive effect on endothelial dysfunctions, an *in vitro* wound closure (scratch assay) was performed to monitor the rate of cell migration into the denuded area of a confluent monolayer. As shown in Fig. 4G,H, miR-135b-5p or miR-499a-3p accelerated the rates of cell migration compared with miR-NC transfected cells. The migration was enhanced in (miR-135b and miR-499a-3p) transfection cells compared to the single miRNA-transfected cells and the migration difference was a result of the impaired expression of MEF2C (Fig. 4G,H). Moreover, ECs migration regulated by the miRNAs was also tested across Transwell filters. As shown in Fig. 4I,J, the migration rate of HUVECs treated with miR-135b-5p or miR-499a-3p was higher than that of miR-NC treated cells. Correspondingly, the migration rate of ECs treated with the two miRNAS was higher than cells treated with single miRNAs, and the increase in cell migration caused by the two miRNAs was offset in (miR-135b-5p + miR-499a-3p + pcDNA3.1-MEF2C) cells. Together, these data indicated that both miR-135b-5p and miR-499a-3p could jointly promote ECs proliferation and migration by directly repressing *MEF2C*.

### MiR-135b-5p and MiR-499a-3p Targeted MEF2C Expression and Enhanced Proliferation and Migration of VSMCs

**Figure 5:**
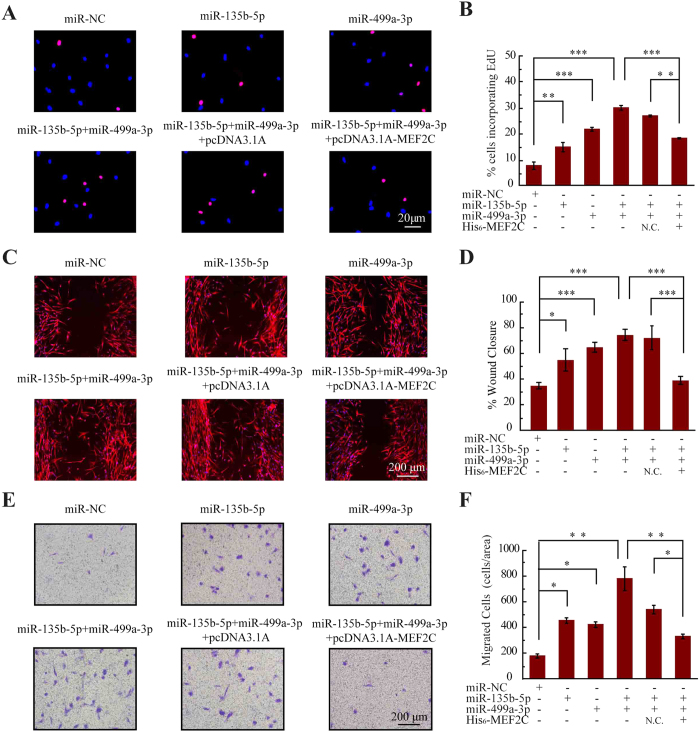
MiR-135b-5p and miR-499a-3p promote smooth muscle cell proliferation and migration **(A)** Photoimages of EdU incorporation assay of VSMCs that were treated with miRNAs or rescue plasmid. Results are representative data from three independent experiments. **(B)** Proliferation rates of VSMCs that were treated with miRNAs or rescue plasmid. Results are presented as means ± SD of three independent experiments. *P < 0.05; ** *P* < 0.01. **(C)** Photoimages of scratch assay of VSMCs that were treated with miRNAs or rescue plasmid. Results are representative data from three independent experiments. **(D)** Migration rates of VSMCs that were treated with miRNAs or rescue plasmid. Results are presented as means ± SD of three independent experiments. *P < 0.05; ***P < 0.001. **(E)** Photoimages of transwell assay of VSMCs that were treated with miRNAs or rescue plasmid. Results are representative data from three independent experiments. **(F)** Migration rates of VSMCs that were treated with miRNAs or rescue plasmid. Results are presented as means ± SD of three independent experiments. *P < 0.05; **P < 0.01.

In the pathogenesis of atherosclerosis, ECs dysfunction has been implicated as an early step and the following VSMCs proliferation and migration is particularly important associated with atherosclerotic progression^4^,^30^. As shown in Fig. 5A,B, proliferation rate of VSMCs transfected with miR-135b-5p mimic or miR-499a-3p mimic was significantly increased by approximately 1.9-fold and 2.7-fold, respectively. Particularly, the proliferation rate was enhanced in both miR-135b and miR-499a-3p transfection cells. The *in vitro* wound closure and transwell assays showed that transfection of miR-135b/-499a mimics significantly increased the migratory ability of VSMCs when compared with control cells (Fig. 5C–F). Furthermore, high-expression of MEF2C restored the proliferation and migration enhancement induced by the two miRNAs. Thus, miR-135b-5p/499a-3p upregulation could stimulate VSMCs proliferation and migration by directly repressing *MEF2C*. In summary, HUVECs and VSMCs underwent profound phenotypic modulation, adopting more proliferative and migratory phenotype after transfection with miR-135b-5p and miR-499a-3p, and the responses of HUVECs and VSMCs were regulated by the miRNAs-mediated MEF2C suppression.

### MEF2C Expression in Human Atherosclerotic Plaques

Increasing evidences suggest that circulating miRNAs protect against degradation by MVs, apoptotic bodies, or lipids and could be taken up by recipient cells, thus serving as signaling molecules mediating intercellular communication^24^,^31^,^32^. Of note, it has been reported that serum miRNAs are more commonly found in MVs, whereas plasma miRNAs are primarily associated with argonaute 2 (Ago2) or high-density lipoprotein (HDL)-associated proteins^32^. Supporting the carrier roles of MVs for the two miRNAs, circulating miR-135b-5p and -499a-3p was shown to be secreted by tumor cells, packaged into MVs and then directly delivered to ECs^16^. We also obtained MVs from serum samples of atherosclerotic CAD patients by sequential centrifugation^33^ and found miR-135b-5p and miR-499a-3p were mainly stored in MVs (Supplementary Fig. S3).

**Figure 6:**
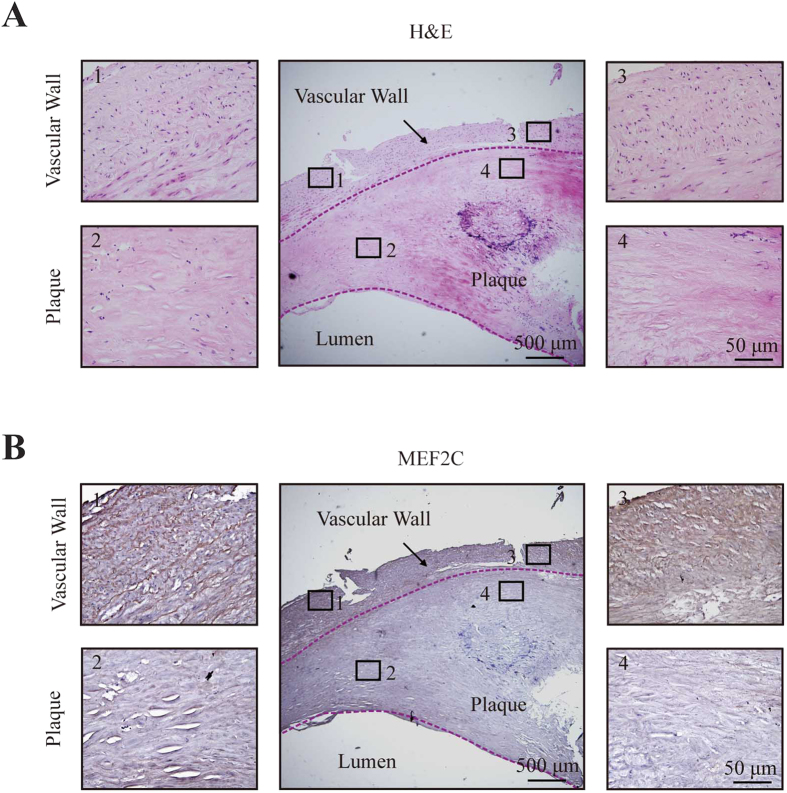
MEF2C protein expression by immunostaining in aortic plaques. **(A)** Representative cross-section of human atherosclerotic plaque with H&E staining (middle photo). Scale bar equals 500 μM. The 1–4 photos were amplification of the four sections from the respective positions (1–4) on the middle photo. Scale bar equals 50 μM. **(B)** Representative cross-section of human atherosclerotic plaque with MEF2C protein staining (middle photo). Scale bar equals 500 μM. The 1–4 photos were amplification of the four sections from the respective positions (1–4) on the middle photo. Scale bar equals 50 μM. The photos showed downregulated MEF2C protein levels in plaques compared with the corresponding vascular wall.

Although the stimuli that trigger miRNA secretion are unclear, accumulating evidences showed that human atherosclerotic plaque contains large amounts of MVs released following cell activation or apoptosis^34^,^35^,^36^. Moreover, MVs isolated from human atherosclerotic lesions originate from multiple cells, including macrophages, lymphocytes, erythrocytes, and smooth muscle cells 34,35. Moreover, it has been demonstrated that plaque MVs dock on nonactivated ECs and contribute to the progression and development of atherosclerotic lesions^36^,^37^. Given that the two miRNAs exist mainly in MVs accumulated in plaque, we hypothesized that miR-135b-5p and miR-499a-3p might be packaged into MVs from atherosclerotic plaques, secreted to circulating systems and taken by ECs and/or VSMCs of vascular wall with MEF2C repression. To address this issue, we first detected the *in situ* hybridization of the miRNAs in human atherosclerotic plaques, but the poor hybridization efficiency precluded the analysis of the two miRNAs expression levels. Because we were not able to detect protein expression in lumen, immunohistochemical staining was performed to compare MEF2C protein levels in plaque with that in vascular wall (Fig. 6). As shown in Fig. 6A,B, MEF2C protein levels were significantly downregulated in human atherosclerotic plaques compared with the corresponding vascular wall. Taken together, the results indicated that miR-135b-5p and miR-499a-3p might be associated with MVs in plaques, and delivered into ECs and VSMCs to repress MEF2C expression.

## Discussion

In the present study, a distinct serum-based miRNA expression pattern in atherosclerotic CAD patients was demonstrated. In particular, 2 miRNAs, miR-135b-5p and miR-499a-3p, were found to be highly expressed in atherosclerotic patients. Given that circulating miRNAs are a potential key feature of atherosclerosis-associated dysfunction of gene regulatory networks^26^,^33^,^38^, our study focused on the potential cellular properties regulated by miR-135b-5p and miR-499a-3p. The *MEF2C*-encoded mRNA contains a 3′-UTR element that is partially complementary to miR-135b-5p and miR-499a-3p, indicating that the miRNAs might directly target the corresponding sites. Interestingly, the *MEF2C*-encoded mRNA contains a 3′-UTR element that is also partially complementary to miR-21 and miR-98, which were downregulated in the miRNA expression profile. It has been demonstrated that the reduction of miR-98 might increase MEF2C protein levels (Supplementary Fig. S4). It would be interesting to study the regulations by which miR-98 target *MEF2C* in further studies; however, in the present study, we focused on investigation of the elevated miRNAs for their potential roles in the release of MVs induced by cell activation.

In atherosclerosis, ECs activation triggers platelet aggregation, leukocyte adhesion and VSMCs proliferation^1^. The VSMCs proliferation within atherosclerotic lesions contributes significantly to luminal narrowing and to plaque instability^1^,^39^. Therefore, proliferation and migration of ECs and VSMCs have been implicated to be critical in the progression of atherosclerotic lesions^1^,^36^. Our exploration to assess the biological functional roles of the two miRNAs revealed interesting and encouraging results. We showed that both miR-135b-5p and miR-499a-3p directly regulated *MEF2C* and had additive enhancement effects on proliferation and migration of ECs and VSMCs. MEF2C, as the third member of the MEF2 transcription factor family (MEF 2A, -B, -C, and -D), plays a important role in modulating cell phenotype^13^,^14^,^15^. It has been reported that *MEF2C* is involved in proliferation of VSMCs^40^ and migration of human retinal endothelial cells^13^. Here, repression of *MEF2C* caused by elevation of miR-135b-5p and miR-499a-3p leaded to a considerable increase in proliferation and migration. The presence of another extracellular miRNA, miR-150, supports these findings. Secreted miR-150, which is increased in the plasma of atherosclerotic patients, is taken up by endothelial cells and regulates cellular migration by repressing the c-Myb gene^33^. Thus, abnormally circulating miRNA expression is likely to contribute to the recipient endothelial cell dysfunction involved in the pathogenesis of atherosclerosis. However, circulating miRNAs that are not endogenously expressed in the recipient cell might be especially appropriate for demonstrating that transferred miRNA is essential for directly modulation in the target cell^8^. Therefore, ECs might be a better selection than VSMCs, because ECs expresses minimal endogenous miR-135-5p and miR-499a-3p, and endothelial dysfunction is particularly a driving force in the initiation and development of atherosclerosis^1^.

Next, there are several other significant factors that influence ECs and VSMCs migration in atherosclerosis, such as HDLs and statin treatment. Notably, the atheroprotective effect of HDL on endothelial cell migration has been reported^41^. We then confirmed the dose-dependent effect of mevastatin on migration^42^ and demonstrated levels of MEF2C increased with both lower and higher concentrations of mevastatin (Supplementary Fig. S5). Thus, further investigation of the influencing factors of miR-135-5p and miR-499a-3p and their target genes is needed to delineate the full range of physiological functions of the 2 miRNAs.

**Figure 7:**
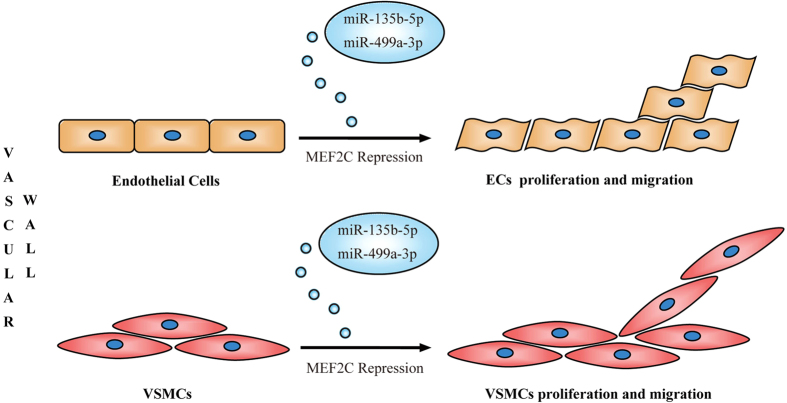
Putative mechanism by which miR-135b-5p and miR-499a-3p might target MEF2C to promote cell proliferation and migration. See the discussion section for further details.

Furthermore, increasing evidences suggest that circulating miRNAs are taken up by recipient cells as signaling molecules of gene regulatory networks^24^,^25^,^26^. Although the stimuli that trigger miRNA secretion are unclear, it has been shown that human atherosclerotic plaque contains large amounts of MVs released following cell activation or apoptosis^34^,^35^,^36^. Several findings further confirm that MVs dock on nonactivated endothelial cells and contribute to the progression and development of atherosclerotic lesions^36^,^37^. Together with our data, there is a plausible explanation for the causes and consequences of circulating miR-135b-5p and miR-499a-3p in atherosclerotic CAD patients (Fig. 7). MiR-135b-5p and miR-499a-3p might be associated with MVs in atherosclerotic lesion with repression of MEF2C. Some of the MV-associated miRNAs could be secreted into circulating systems. Finally, the MVs might dock on ECs and /or VSMCs and the two miRNAs could be taken by the cells as molecular signals to facilitate atherosclerotic development by enhancing proliferation and migration. However, MVs isolated from human atherosclerotic lesions originate from multiple cells, including macrophages, lymphocytes, erythrocytes, and smooth muscle cells^34^,^35^. Thus, the cell origins of secreted miR-135b-5p and miR-499a-3p remain unknown because no general mechanism for MVs formation has been demonstrated.

In conclusion, these results demonstrated a serum-based miRNA profile for atherosclerotic CAD patients and indicate circulating miRNAs could regulate target gene expression and recipient cell function. The elevated miR-135b-5p and miR-499a-3p directly targeted *MEF2C* gene together, and had additive effect on increased proliferation and migration of ECs and VSMCs. These findings may reveal important insights into the role of circulating miRNAs in the pathogenesis of atherosclerosis, and further suggest that miR-135b-5p and miR-499a-3p serve as a novel therapeutic target for atherosclerosis. However, a large number of specific miRNAs might be involved in atherosclerotic disease. Thus, investigation of additional circulating miRNAs and their functional roles in atherosclerosis are necessary to offer insights into the pathophysiology and potential role in targeted therapy for coronary artery disease.

## Methods

### Study Population

The complete details of the entire study design and procedures involved were in accordance with the Declaration of Helsinki. Informed written consent was obtained from all CAD and control subjects and the study protocol was approved by the Ethics Review Committee for Human Studies of the Shandong University School of Medicine. The methods used were carried out in accordance with approved guidelines and regulations.

Ninety atherosclerotic patients with symptoms of angina pectoris were included in the study after angiographic documentation of CAD. Fifty healthy volunteers without any evidence of CAD, diabetes mellitus, or inflammatory disorders were enrolled from the Shizhong District in Eastern Jinan and served as the control group. Fifteen non-atherogenic cardiovascular patients (3 with cardiogenic shock, 5 with heart failure, and 7 with ventricular arrhythmia) served as another control group in the independent validation of miR-135b-5p and miR-499a-3p expressions. Atherosclerotic plaques were collected from patients undergoing vascular surgery or at autopsy.

### Serum Collection, RNA Isolation, and MiRNA Profile

Five-milliliter peripheral blood samples were separated into serum fractions by centrifugation at 1000 g for 10 min. Serum RNA was harvested with the TRI Reagent BD (Sigma-Aldrich Co., LLC, St. Louis, MO, USA) and the RNeasy mini kit (Qiagen, Inc., Valencia, CA, USA) according to the manufacturers’ instructions. The miRNA microarray profiling was conducted by KangChen Bio-tech, Inc. (Shanghai, China). The miRNA expression profiling from the serum samples was performed using the miRCURY LNA Array (version. 11.0, Exiqon Inc., Woburn, MA, USA) system (Exiqon, Inc., Woburn, MA, USA). RNA samples were labeled using the miRCURY Hy3/Hy5 Power labeling kit (Exiqon Inc., Woburn, MA, USA) and hybridized on the miRCURY LNA Array station. Scanning was performed with the Axon GenePix 4000B microarray scanner (Molecular Devices, LLC, Sunnyvale, CA, USA). GenePix Pro version 6.0 was used to read the raw data of the images. The intensity of the signal was calculated after background subtraction, and replicated spots in the same image were averaged to obtain the median intensity. The median normalization method was used to obtain normalized data (foreground minus background divided by median). The median represents the 50^th^ of miRNA intensity, which is >50 in all samples after background correction. The significance of the results was determined using fold change and *t* tests. The threshold value for significance used to define upregulation or downregulation of miRNAs was a fold change >2 and *p*-value <0.05. The miRNAs selected for investigation in this study were further filtered on the basis of expression levels and previously published data.

### Detection and Quantification of MiRNAs by qRT-PCR

To confirm the findings obtained by analyzing the miRNA profiling, real-time quantitative RT-PCR (qRT-PCR) analysis was performed using an ABI 7500 system (Applied Biosystems, Foster City, CA). The relative expression level of miRNAs was normalized to that of the internal control hsa-miR-93 using the 2^−ΔΔCt^ cycle threshold method. The reverse transcription primers and the primer sets specific for amplification of miR-135b-5p and miR-499a-3p were as follows:

miR-499a-3p RT, 5′-GTCGTATCCAGTGCGTGTCGTGGAGTCGGCAATTGCACTGGATACGACAGCACA-3′;

miR-499a-3p F, 5′-GGGAACATCACAGCAAGTC-3′;

miR-499a-3p R, 5′-CAGTGCGTGTCGTGGAG3′;

miR-135b-5p RT 5′-GTCGTATCCAGTGCGTGTCGTGGAGTCGGCAATTGCACTGGATACGACTCACAT-3′;

miR-135b-5p F, 5′-GGTATGGCTTTTCATTCCT-3′;

miR-135b-5p R, 5′-CAGTGCGTGTCGTGGAGT3′.

### 3′-UTR Luciferase Reporter Assays

The *MEF2C* 3′-UTR luciferase reporter construct was made by amplifying the human *MEF2C* mRNA 3′-UTR sequence and cloning it to the 3' end of a firefly luciferase reporter gene in the pmirGLO dual luciferase vector (Promega Corp., Madison, WI, USA). Site-directed mutagenesis of a miR-135b-5p or miR-499a-3p target site of *MEF2C* mRNA was carried out using a site-directed gene mutagenesis kit (Beyotime Institute of Biotechnology, Beijing, China). The specific mutagenesis primers were as follows:

miR-499a-3p F, 5′-TGTGCCATCTGCCGTTGGGTCGTCACTTTTATGG-3′;

miR-499a-3p R, 5′-CCATAAAAGTGACGACCCAACGGCAGATGGCACA-3′;

miR-135b-5p F, 5′-TGAAAATATGAAGAAATAGTTGTATTAGTTTTTTAACCTGC-3′;

miR-135b-5p R, 5′-GCAGGTTAAAAAACTAATACAACTATTTCTTCATATTTTCA-3′.

HEK293 cells were cotransfected with 20 ng luciferase reporter plasmid and the indicated miRNAs (final concentration, 25 nmol/L) using Lipofectamine 2000 (Invitrogen, Carlsbad, CA, USA) according to the manufacturer’s instructions.

### Cell Culture, Transfection and Immunological Procedures

HEK293, HUVECs and human aortic VSMCs were purchased from ATCC. For overexpression of miRNAs, 100 pmol/L of miR-135b-5p mimic, miR-499a-3p mimic or negative control mock-miRNA (GenePharma, Beijing, China) was used. For knockdown of miRNAs, 100 pmol/L of miR-135b-5p inhibitor, miR-499a-3p inhibitor or negative control miRNA inhibitor (GenePharma, Beijing, China) was used. Cells were harvested 72 hours after transfection. Construction of pcDNA3.1/myc-His-MEF2C was accomplished by subcloning a PCR-amplified in frame MEF2C fragment into the pcDNA 3.1-myc-His A vector between BamHI and XbaI sites using HEK293 cDNA as a template.

Antibodies against the following proteins were purchased: anti-MEF2C (SC13266, polyclonal antibody produced in goat), anti-β-actin (SC-8432, monoclonal antibody produced in mouse), anti-α-tubulin (SC-53646, monoclonal antibody produced in mouse) (Santa Cruz Biotechnology, Santa Cruz, CA) and anti-MEF2C for IHC-P (ab191428, monoclonal antibody produced in rabbit) (Abcam, Cambridge, MA). Immunoblotting and immunofluorescence staining were performed as described previously^43^. For immunohistochemisty examination of the tissue samples, hematoxylin and eosin (H&E) staining was performed according to standard procedures. Sections were sequentially blocked with 3% H_2_O_2_ for 10 min, 10% normal serum matching the host of the secondary antibody for 60 min at room temperature. Primary antibody against human MEF2C (ab191428 from Abcam, Cambridge, MA) was used for immunostaining. Secondary antibody (goat anti-rabbit) was used at 1:100 dilutions. Sections were developed by DAB substrate and counterstained with hematoxylin for miscroscopic visualization.

### EdU Incorporation and Migration Assays

EdU (Cell-Light^TM^ EdU Cell Proliferation Detection Kit, Guangzhou RiboBio, China) was added at 100 μM and the cells were cultured for an additional 2 h. After the removal of EdU-containing medium, the cells were fixed with 4% paraformaldehyde at room temperature for 30 min, washed with glycine (2 mg/ml) for 5 min in a shaker, treated with 0.2% Trion X-100 for 10 min, washed with PBS twice. Click reaction buffer (Tris-HCl, pH 8.5, 100 mM; CuSO_4_, 1 mM; Apollo 550 fluorescent azide, 100 μM; ascorbic acid, 100 mM) was then added. After 20 min, the cells were washed with 0.5% Triton X-100 for three times, stained with 4′,6-diamidino-2-phenylindole (DAPI) for 10 min at room temperature, washed with 0.5% Triton X-100 for five times, and finally, immersed in 150 μL PBS and examined under a fluorescence microscope.

The cell migration ability was tested in Transwell Permeable Supports (Corning Incorporated, Lowell, MA, USA). After infection with the indicated miRNAs, the cells were suspended in serum-free Dulbecco’s modified eagle’s medium (DMEM) at a concentration of 10^5^/mL, and 200 μL suspensions were placed in the upper chamber of the Transwell. The chamber was then transferred to a well containing 500 μL media containing 10% fetal bovine serum. Cells were incubated for 5 h at 37 ^°^C. Cells in the top well were removed by wiping the top of the membrane with cotton swabs. The membranes were then stained and the remaining cells were counted. The contents of 4 high-powered fields were counted for each membrane.

### Statistical Analysis

All continuous variables are expressed as means ± standard deviation (SD), unless stated otherwise. Categorical variables were compared using the Chi squared (*x*^*2*^) test, and continuous variables were compared using the Student’s *t*-test. All tests were performed 2-sided and a significance level of *P* < 0.05 was considered to indicate statistical significance (*P < 0.05, **P < 0.01, ***P < 0.001 and ^#^*P* < 0.0001). GraphPad Prism 5.0 (GraphPad software Inc., San Diego, CA, USA) was used for all statistical analyses. All photo images of Western Blotting, Edu incorporation, migration assays and immunohistochemical staining are representative of at least three independent experiments.

## Additional Information

**How to cite this article**: Xu, Z. *et al.* MiR-135b-5p and MiR-499a-3p Promote Cell Proliferation and Migration in Atherosclerosis by Directly Targeting MEF2C. *Sci. Rep.* **5**, 12276; doi: 10.1038/srep12276 (2015).

## Supplementary Material

Supplementary Information

## Figures and Tables

**Table 1:** Properties of microRNAs differentially expressed in atherosclerotic CAD patients compared with healthy control subjects.

**miRNA**	**Fold change**	***P* value**	**Chromosome location**	**Potential targets**
Upregulated				
miR-20b-5p	4.73	<0.0001	Xq26.2	*MLL5, THRA*
miR-138-1-3p	3.79	<0.0001	3p21.32	*PCNT, BMP*
miR-499a-3p	18.97	<0.0001	20q11.22	*MEF2C, FOXN3*
miR-218-2-3p	10.37	<0.01	5q34	*GLCE, TUB*
miR-142-5p	7.47	<0.01	17q22	*AFF4, MKL2*
miR-147b	6.12	<0.001	9q33.2	*IGF2, INS*
miR-135b-5p	6.43	<0.001	1q32.1	*MEF2C, FOXN3*
miR-302f	14.8	<0.001	18q12.1	*GGCX, PROSC*
ebv-miR-BART7-5p	4.43	<0.001		
miR-155	1.21	0.414	21q21.3	*UACA, ZIC3*
Downregulated				
miR-21	0.135	<0.0001	17q23.1	*NFIB, PPARA*
miR-98	0.13	<0.01	Xp11.22	*PAPPA, PTPRD*
miR-379	0.132	<0.0001	14q32.31	*FIGN, HLCS*
