# Supplementary material for: MiR-135b-5p and MiR-499a-3p Promote Cell Proliferation and Migration in Atherosclerosis by Directly Targeting MEF2C
Source: Sci Rep. 2015 Jul 17;5:12276. doi: 10.1038/srep12276 (PMC4505325; doi:10.1038/srep12276)

SUPPLEMENTARY MATERIAL FOR

MiR-135b-5p and MiR-499a-3p Promote Cell Proliferation and Migration in Atherosclerosis by Directly Targeting MEF2C

Zhiliang Xu[1], Yeming Han[2], Jiying Liu[3], Fan Jiang[4], Huili Hu[1], Yan Wang[1], Qiji Liu[1], Yaoqin Gong[1] and Xi Li[1,*]

From the [1]Key Laboratory of Experimental Teratology, Ministry of Education

Institute of Medical Genetics, School of Medicine, Shandong University

Jinan, Shandong 250012, China

[2]Department of Cardiology, Shandong University Qilu Hospital, Jinan, Shandong 250012, China

[3]Department of Interventional Treatment, Shandong Provincial Hospital Affiliated to Shandong University, Jinan, Shandong, 250021, China

[4] Key Laboratory of Cardiovascular Remodeling and Function Research, Ministry of Education and Ministry of Health, Shandong University Qilu Hospital, Jinan, Shandong 250012, China

I. SUPPLEMENTARY TABLES

CAD: coronary artery disease; BMI: body mass index; MI: myocardial infarction; PCI: percutaneous coronary intervention; ACVB: aortocoronary venous bypass; PTA: percutaneous transluminal angioplasty. Age is given as mean ± S.D., and other values are number of individuals with percentage (%) in parentheses. *P<0.05; ** P<0.01 and *** P<0.005.

Supplementary Table S1: Clinical characteristic of atherosclerotic patients and healthy controls included in the miRNA profiling

Variables	Patients (n=13)	Controls (n=5)	*P* Value
Age (years)	59.26±5.35	30.27±3.79	<0.001***
Male gender, N (%)	6 (46.15%)	2 (40%)	NS
Stable CAD for at least 4 months	13 (100%)	0	<0.005***
Number of vessels (CAD)	I 3 (23.07 %)	I 0	NS
	II 8 (61.53%)	II 0	NS
	III 2 (15.38%)	III 0	NS
Hypertension	8 (61.53%)	0	<0.025*
Diabetes mellitus	3 (23.08%)	0	NS
Active smoker	3 (23.08%)	0	NS
Obesity (BMI> 25 kg/m^2)	2 (15.38%)	0	NS
MI/PCI/ACVB/PTA/Stroke	13 (100%)	0	<0.005***
Beta-blocker	13 (100%)	0	<0.005***
Aspirin/Clopidogrel	13 (100%)	0	<0.005***
ACE-Inhibitor/ATRB	10 (76.92%)	0	<0.005***
Diuretics	2 (15.38%)	0	NS
Statin therapy	11 (84.62%)	0	<0.005***
Total cholesterol (mmol/L)	5.33±1.16	4.88±0.47	NS
LDL cholesterol (mmol/L)	3.56±0.76	2.91±0.49	NS
HDL cholesterol (mmol/L)	1.18±0.21	1.19±0.21	NS
Triglycerides (mmol/L)	1.72±0.46	1.38±0.53	NS

Supplementary Table S2: Clinical characteristic of 77 atherosclerotic patients and 45 healthy controls included in the miRNA profiling

**Variables**	**Patients** (n=77)	**Controls** (n=45)	***P* Value**
Age (years)	54.29±8.64	43.26±4.71	<0.005***
Male gender, N (%)	44 (57.14%)	23 (51.11%)	NS
Stable CAD for at least 4 months	77 (100%)	0	<0.005***
Number of vessels (CAD)	I 30 (38.96 %)	I 0	<0.01**
	II 35 (45.45%)	II 0	<0.005***
	III 12 (15.58%)	III 0	NS
Hypertension	56 (72.71%)	0	<0.005***
Diabetes mellitus	40 (51.95%)	0	<0.005***
Active smoker	36 (46.75%)	9 (20%)	NS
Obesity (BMI> 25 kg/m^2)	15 (19.46%)	3 (6.76%)	NS
MI/PCI/ACVB/PTA/Stroke	77 (100%)	0	<0.005***
Treatments:			
Beta-blocker	77 (100%)	0	<0.005***
Aspirin/Clopidogrel	77 (100%)	0	<0.005***
ACE-Inhibitor/ATRB	56 (72.72%)	0	<0.005***
Diuretics	15 (19.48%)	0	NS
Statin therapy	60 (77.92%)	0	<0.005***
Total cholesterol (mmol/L)	5.34±0.78	4.69±0.47	<0.01**
LDL cholesterol (mmol/L)	3.76±0.37	2.80±0.49	<0.001***
HDL cholesterol (mmol/L)	1.19±0.49	1.21±0.31	NS
Triglycerides (mmol/L)	1.82±0.39	1.39±0.67	<0.005***

Supplementary Table S3: Correlation of circulating miRNAs with baseline characteristics (analysis includes patients and health volunteers) by using Spearman analysis

	miR-135b-5p	miR-499a-3p
Age	**R = 0.705 *P* = 0.0001**	**R = 0.676 *P* = 0.0001**
Gender	R = 0.259 *P* = 0.3	R = 0.259 *P* = 0.3
Hypertension	R = 0.409 *P* = 0.092	R = 0.065 *P* = 0.799
Diabetes	R = 0.273 *P* = 0.273	R = -0.102 *P* = 0.687
Active smoker	R = 0.043 *P* = 0.865	R = -0.014 *P* = 0.955
Obesity	R = 0.239 *P* = 0.341	R = 0.034 *P* = 0.893
MI/PCI/ACVB/PTA/Stroke	**R = 0.777 *P* = 0.001**	**R = 0.777 *P* = 0.001**
Beta-bloker	**R = 0.777 *P* = 0.001**	**R = 0.777 *P* = 0.001**
Aspirin/Clopidogrel	**R = 0.777 *P* = 0.001**	**R = 0.777 *P* = 0.001**
ACE-Inhibitor/ATRB	**R = 0.754 *P* = 0.001**	R = 0.409 *P* = 0.092
Diuretics	R = -0.34 *P* = 0.893	R = 0.341 *P* = 0.166
Statin therapy	R = 0.406 *P* = 0.094	**R = 0.494 *P* = 0.037**

Red indicates significant correlations; gray background indicates no significant correlation.

Supplementary Table S4: Correlation of circulating miRNAs with baseline characteristics (analysis includes only patients) by using Spearman analysis

	**miR-135b-5p**	**miR-499a-3p**
Age	R = 0.095 *P* = 0.410	R = 0.057 *P* = 0.624
Gender	R = 0.454 *P* = 0.119	R = 0.454 *P* = 0.119
Hypertension	R = -0.042 *P* = 0.891	**R = -0.718 *P* = 0.006**
Diabetes	R = 0.171 *P* = 0.577	R =-0.456 *P* = 0.117
Active smoker	R = -0.293 *P* = 0.332	R =-0.390 *P* = 0.187
Obesity	R = 0.114 *P* = 0.711	R = -0.228 *P* = 0.454
ACE-Inhibitor/ATRB	R = 0.488 *P* = 0.091	R = -0.293 *P* = 0.332
Diuretics	R = -0.342 *P* = 0.253	R = 0.285 *P* = 0.345
Statin therapy	R = -0.513 *P* = 0.073	R = -0.285 *P* = 0.345

Red indicates significant correlations; gray background indicates no significant correlation.

II. SUPPLEMENTARY FIGURE LEGEND

Supplementary Figure S1. Comparison of 13 miRNAs (shown in Table 1) in atherosclerotic patients and in the healthy controls. For comparison, the expression levels of miRNAs in healthy controls were arbitrarily set at 1. The y axes showed arbitrary units representing relative miRNA expression levels. The *P* values were calculated by 2-sided Student *t* test. *$P<0.05$; ** $P<0.01$; ***$P<0.001$; #$P<0.0001$.

Supplementary Figure S2. Comparison of 26 miRNAs in atherosclerotic patients and in the healthy controls. For comparison, the expression levels of miRNAs in healthy controls were arbitrarily set at 1. The y axes showed arbitrary units representing relative miRNA expression levels. The *P* values were calculated by 2-sided Student *t* test. *$P<0.05$; ** $P<0.01$; ***$P<0.001$; #$P<0.0001$.

Supplementary Figure S3. (A) Semiquantitative RT-PCR of miRNAs in MVs isolated from serum of normal human subjects **(B)** Comparison of miRNA expression in MV-free serum and in the MV fraction. For comparison, the expression levels of miRNAs in MV-free serum were arbitrarily set at 1. The y axes showed arbitrary units representing relative miRNA expression levels. Results are presented as means ± SD of three independent experiments (***$P<0.001$).

Supplementary Figure S4. (A) Western blot analysis of MEF2C expression in HEK293 cells transfected with the miRNA mimics. **(B)** Compiled data of MEF2C protein analysis from three independent experiments after transfection of miRNAs is shown. Columns, mean; Bars, ± SD; *$P<0.05$. **(C)** Western blot analysis of MEF2C expression in HEK293 cells transfected with the miRNA inhibitors. **(D)** Compiled data of MEF2C protein analysis from three independent experiments after transfection of miRNAs is shown. Columns, mean; Bars, ± SD; *$P<0.05$, ** $P<0.01$.

Supplementary Figure S5. (A) Photoimages of transwell analysis of HUVECs that were treated with mevastatin (0.1 and 5 µM). Results are representative data from three independent experiments. **(B)** Migration rates of HUVECs that were treated with mevastatin (0.1 and 5 µM).

Results are presented as means ± SD of three independent experiments. *** $P<0.001$. **(C)** Western blot analysis of MEF2C expression in HUVECs treated with 0.1 µM mevastatin. **(D)** Compiled data of MEF2C protein analysis from three independent experiments after treatment with 0.1 µM mevastatin is shown. Columns, mean; Bars, ± SD; *$P<0.05$. **(E)** Western blot analysis of MEF2C expression in HUVECs treated with 5 µM mevastatin. **(F)** Compiled data of MEF2C protein analysis from three independent experiments after treatment with 5 µM mevastatin is shown. Columns, mean; Bars, ± SD; *$P<0.05$.

Supplementary Figure S6. Full-length gels of Fig. 3G.

Supplementary Figure S7. (A) Full-length gels of Fig. 4A. **(B)** Full-length gels of Fig. 4C.

SUPPLEMENTARY FIGURE S1

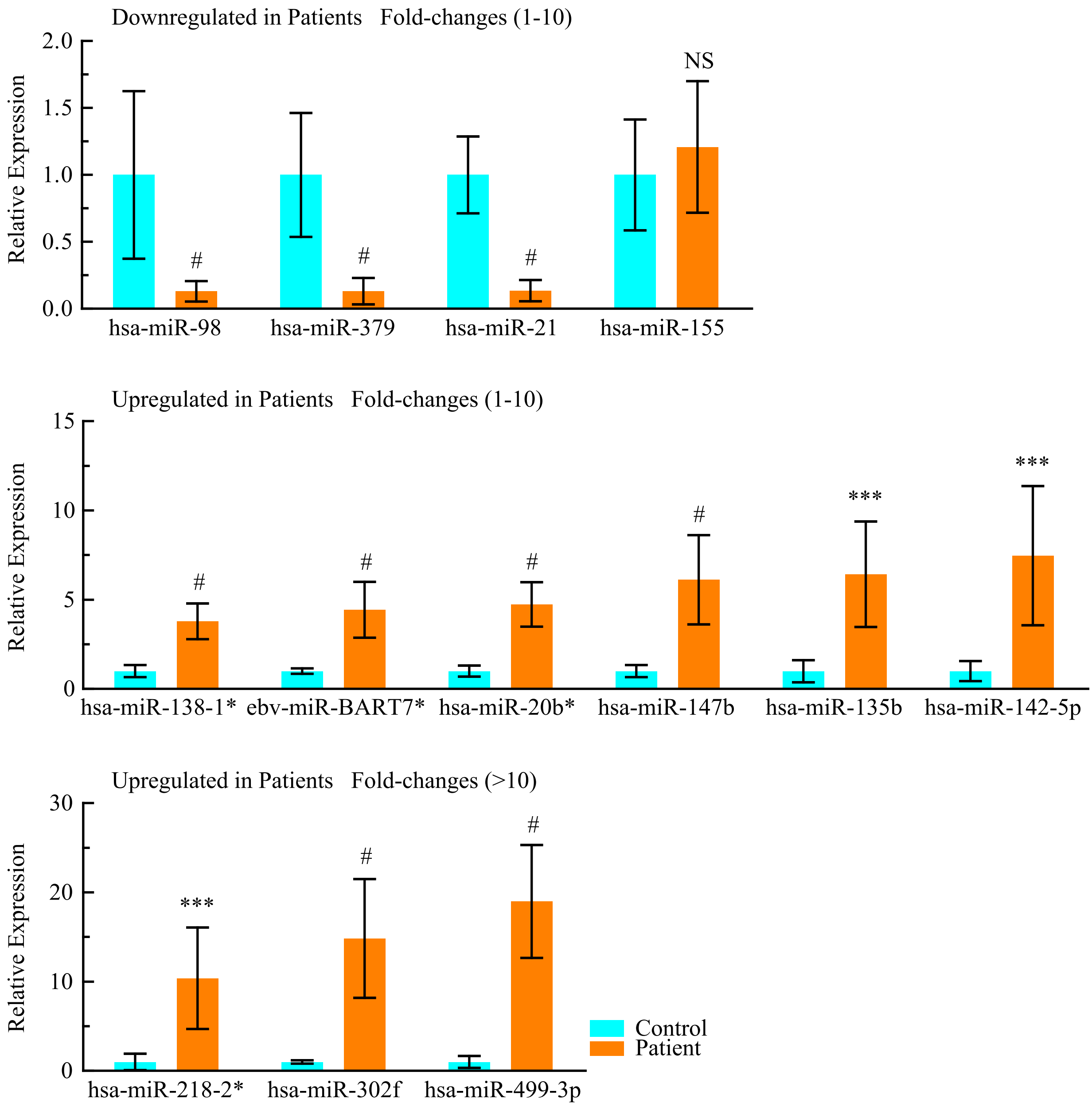

SUPPLEMENTARY FIGURE S2

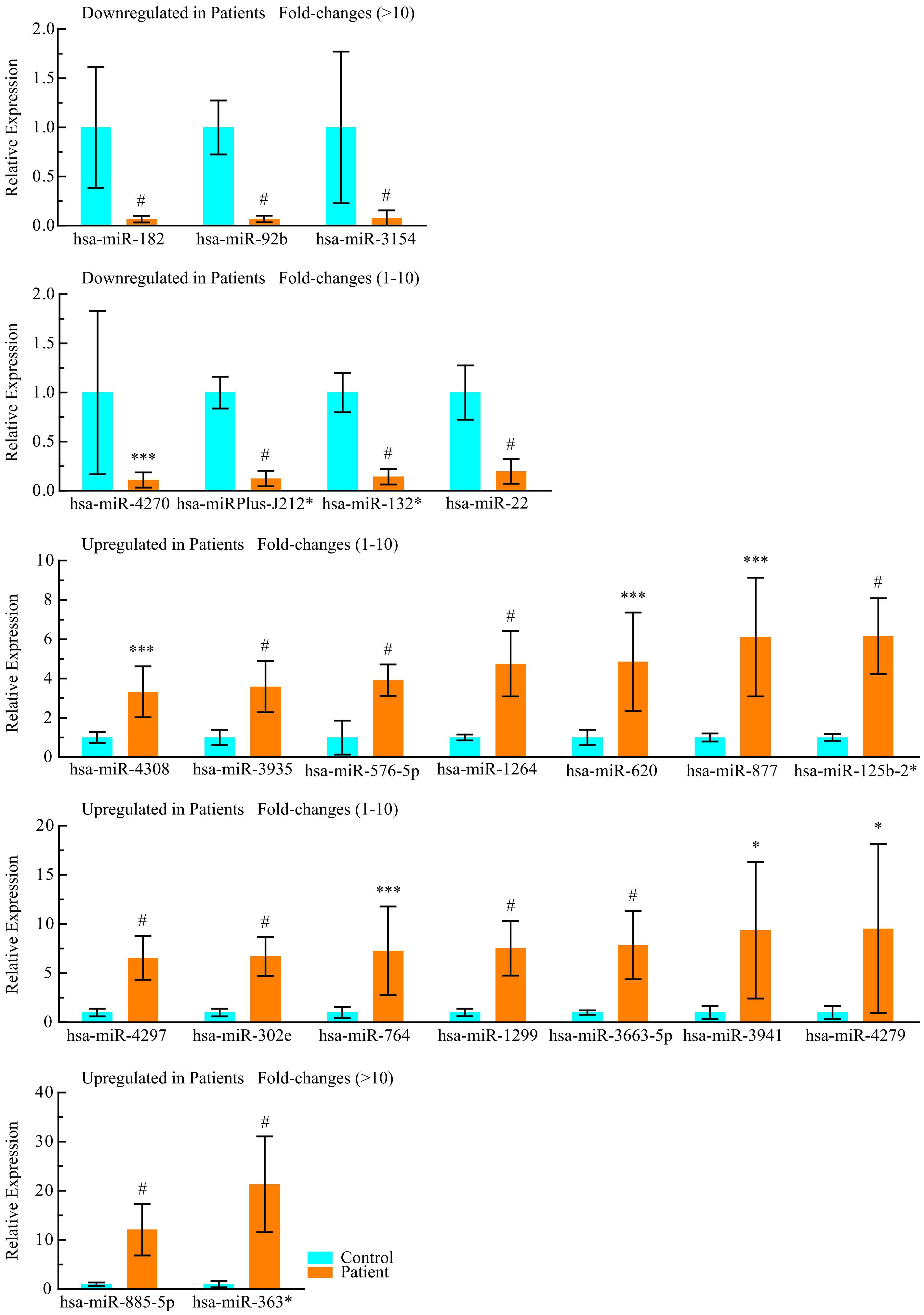

SUPPLEMENTARY FIGURE S3

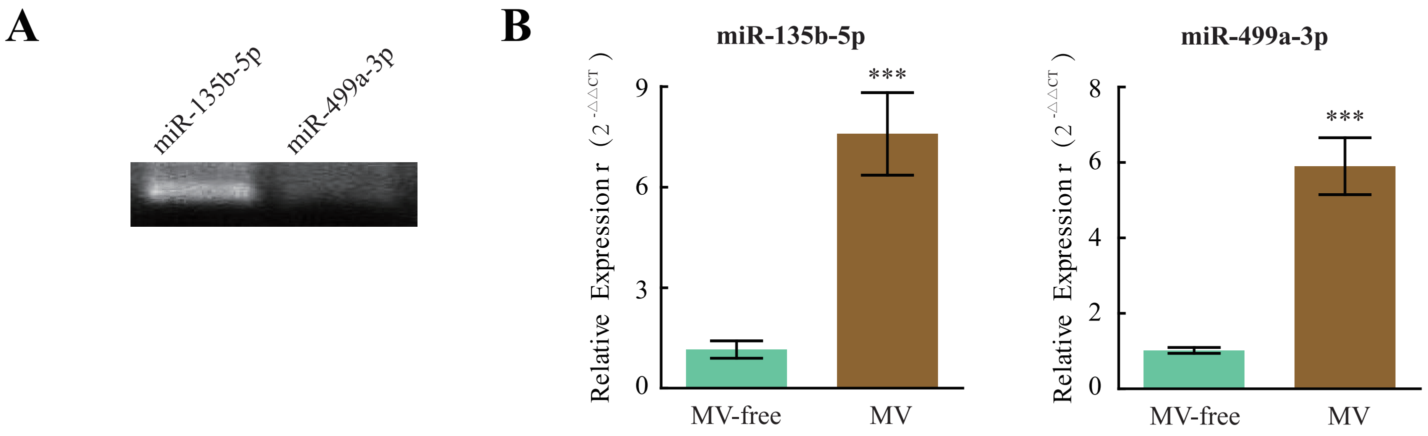

SUPPLEMENTARY FIGURE S4

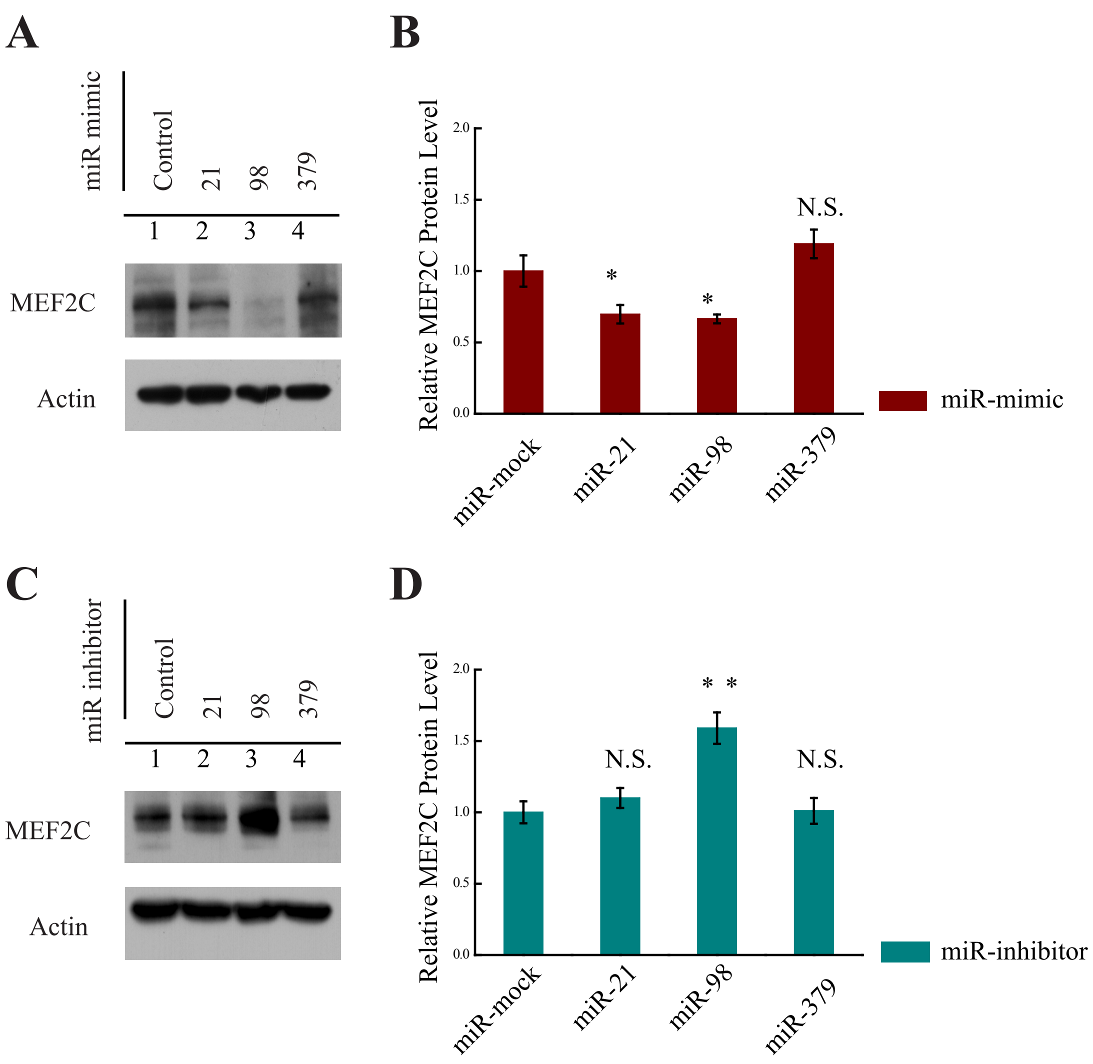

SUPPLEMENTARY FIGURE S5

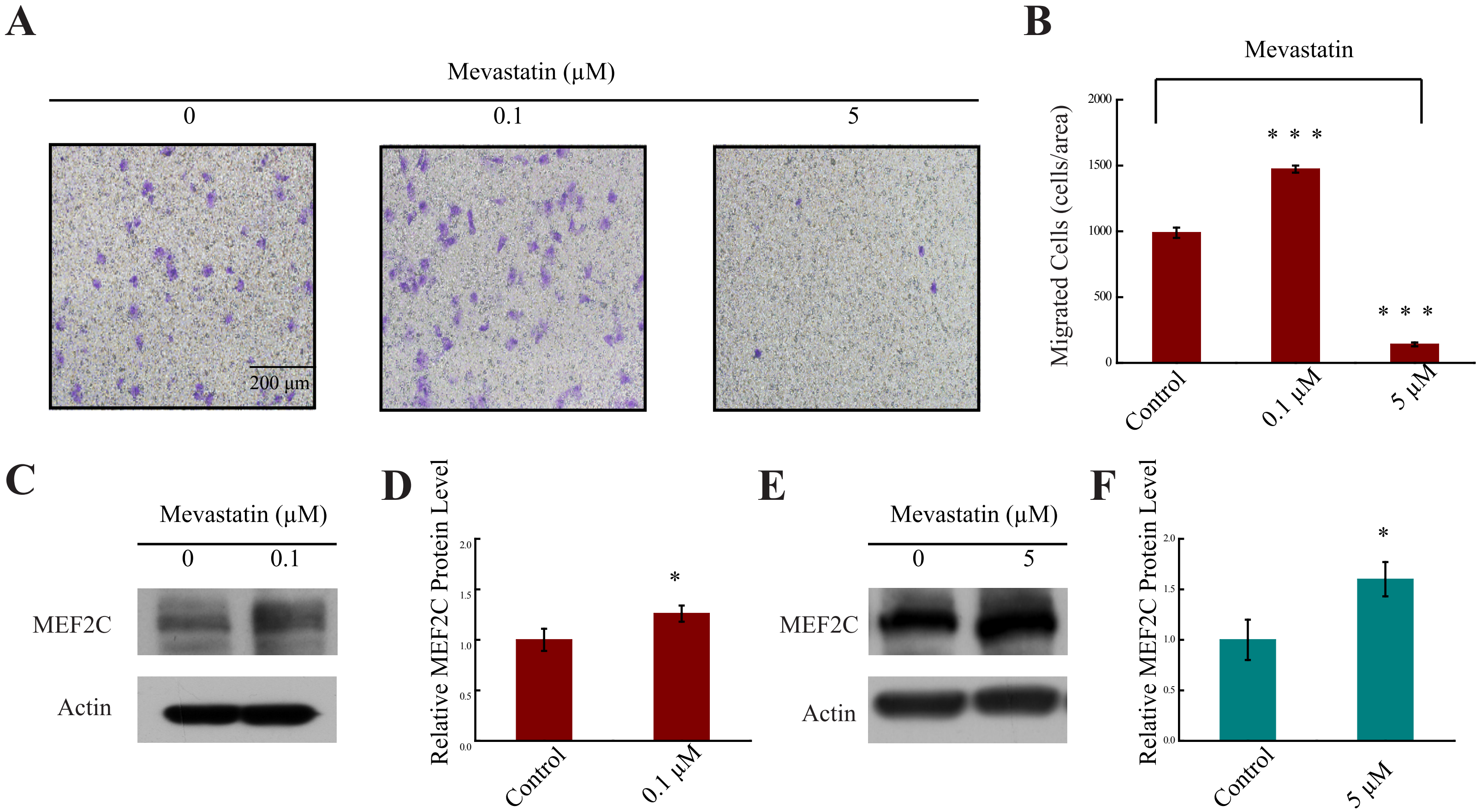

SUPPLEMENTARY FIGURE S6

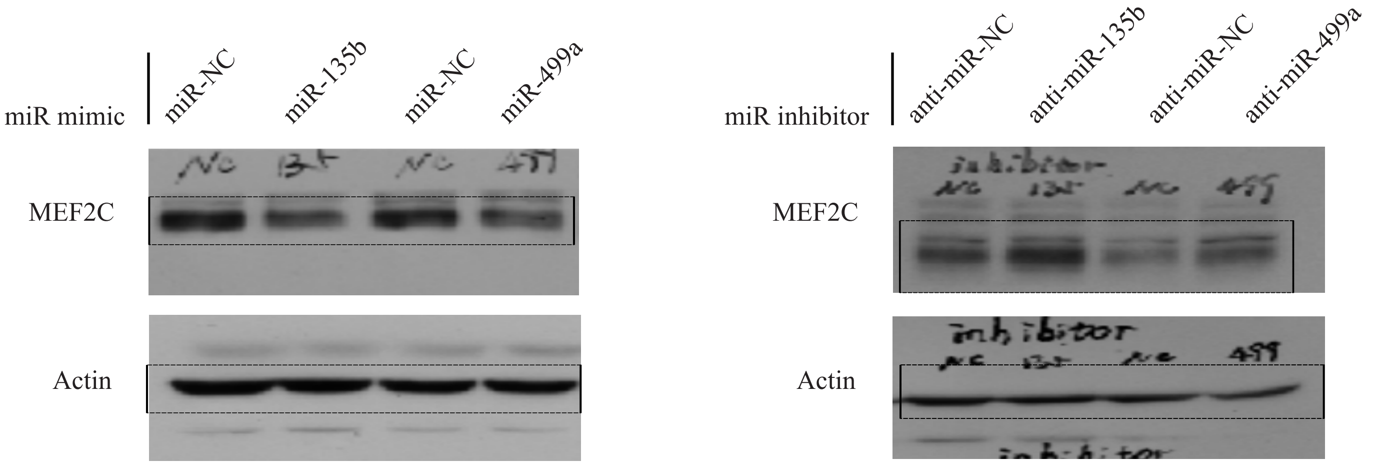

SUPPLEMENTARY FIGURE S7

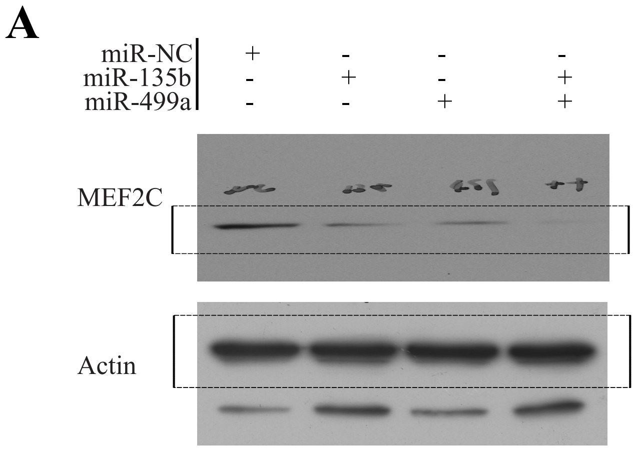

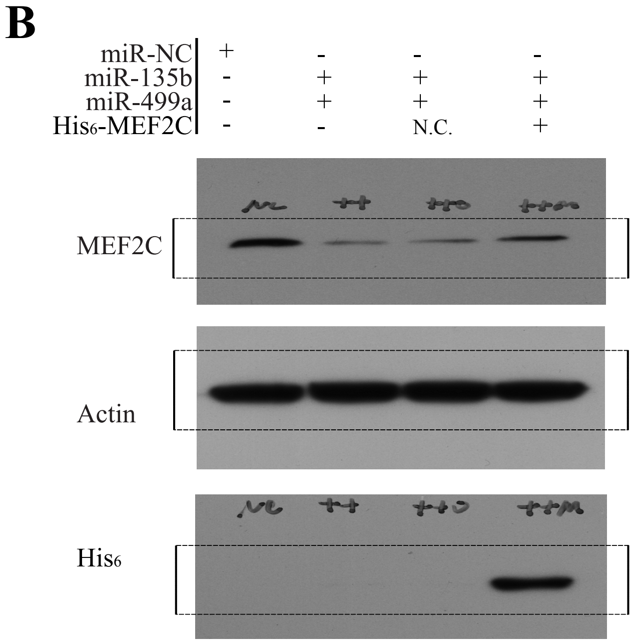

Supplement: Supplementary Information [file srep12276-s1.pdf]
